# Supplementary material for: Yeast Lsm Pro-Apoptotic Mutants Show Defects in Autophagy
Source: Int J Mol Sci. 2023 Sep 5;24(18):13708. doi: 10.3390/ijms241813708 (PMC10530990; doi:10.3390/ijms241813708)
Supplement: Supplementary file 1 [file ijms-24-13708-s001.zip › Supplemental figures_CMFixed_30-08.pdf]

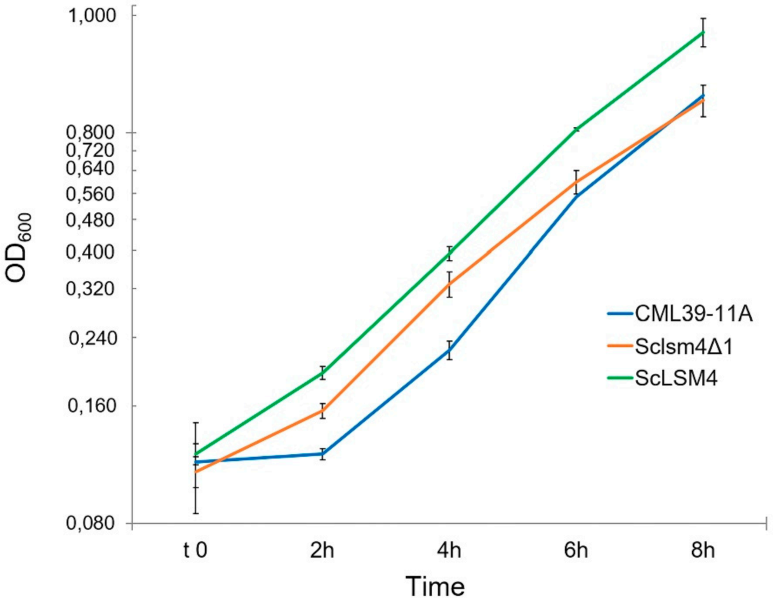

Figure S1. Growth curves relative to MCY4/*Sclsm4Δ1*, MCY4/*ScLSM4*, and the wild-type strain CML39-11A. Strains were exponentially growing in YPD medium, and OD_{600} values were taken every two hours. Error bars represent the standard deviation of three independent biological replicates.

Table S1. *Growth rate (μ) of the strains of interest.*

Strain	Growth rate (μ)
CML39-11A	0,375520
Sclsm4Δ1	0,270481
ScLSM4	0,326280

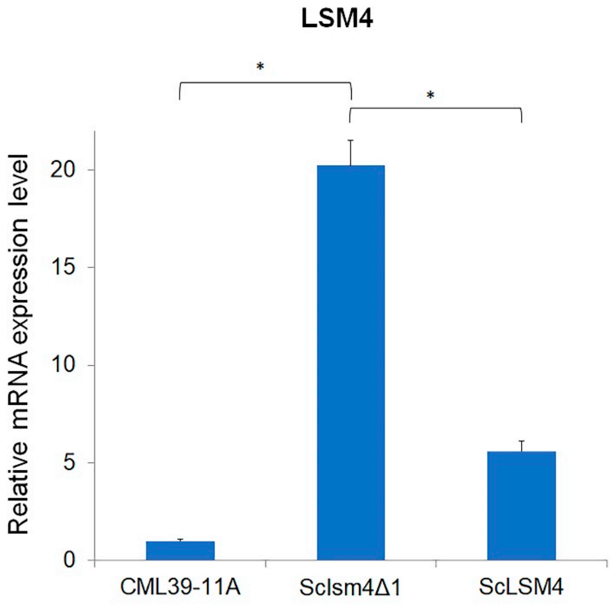

Figure S2. Expression ratio of the *LSM4* gene in the mutant strains MCY4/*Sclsm4Δ1*, MCY4/*ScLSM4* compared to the wild-type strain CML39-11A. RNA was estracted from exponentially growing cells in YPD medium, and the housekeeping gene *TDH3* was used as the calibrator. The mean of the fold change (expressed as $2^{-\Delta\Delta Ct}$) of two biological replicates was plotted. Error bars represent the standard deviation. *p-value<0.05.

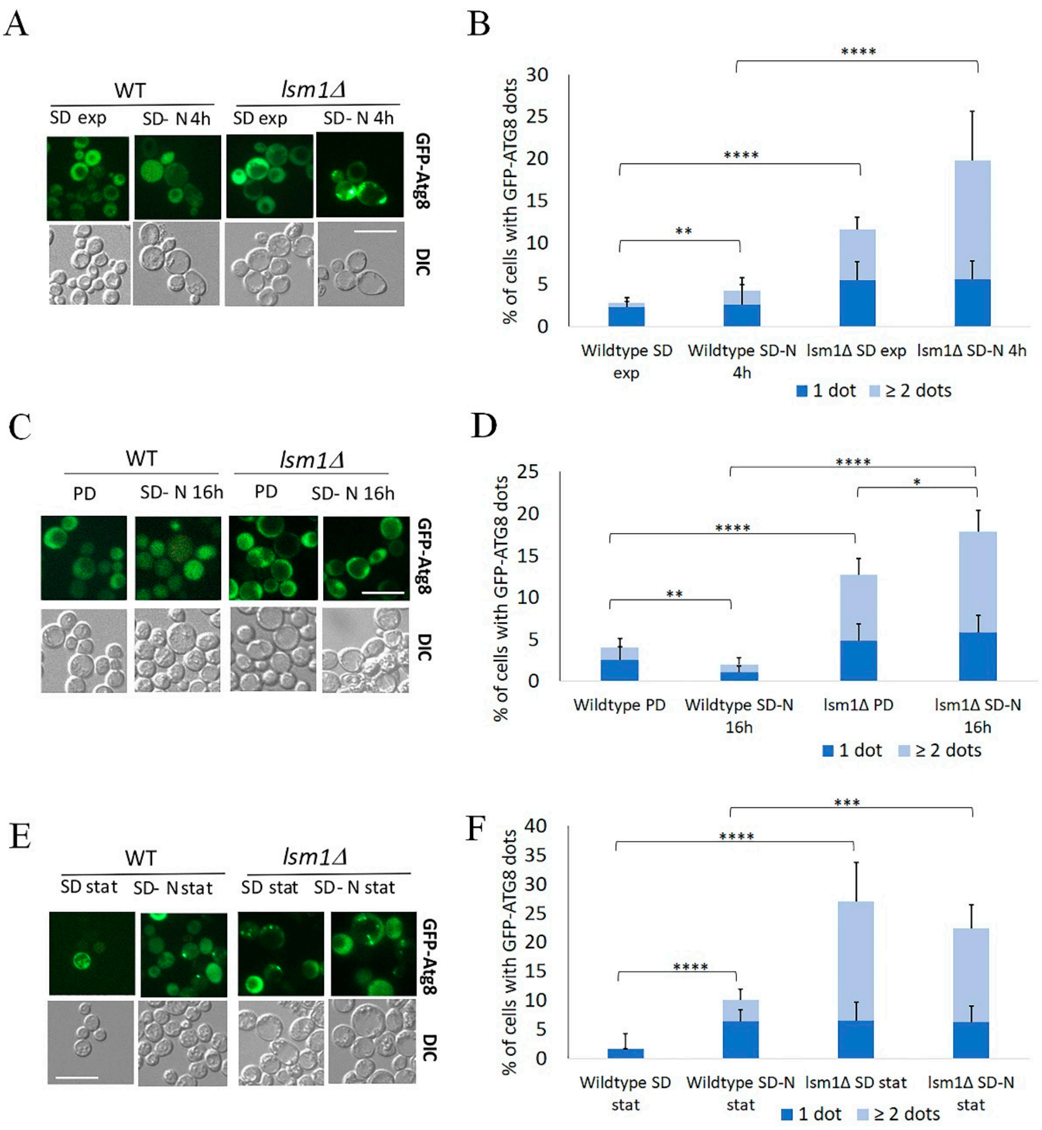

Figure S3. *Lsm1Δ* mutant shows defects in autophagy-related structures transport to the vacuole, as indicated by a higher percentage of GFP-ATG8 dots in the cytoplasm during nitrogen starvation and CLS. Wild-type BMA38 and mutant *lsm1Δ* cells expressing the fusion protein GFP-ATG8 were observed at the fluorescence microscope during the exponential phase in both SD and SD-N medium for 4 hours (A), during the post-diauxic phase (PD) and in SD-N for 16 hours (C) and after 3 days of growth in SD (SD stat) or SD-N (SD-N stat) (E). GFP-Atg8 dots per cell were quantified from three biological replicates ($n \geq 300$ cells), and the mean of cells containing one or ≥2 dots was plotted in (B), (D), and (F). Error bars represent standard deviation. *p-value<0.05 **p-value<0.01 ***p-value<0.001 ****p-value<0.0001

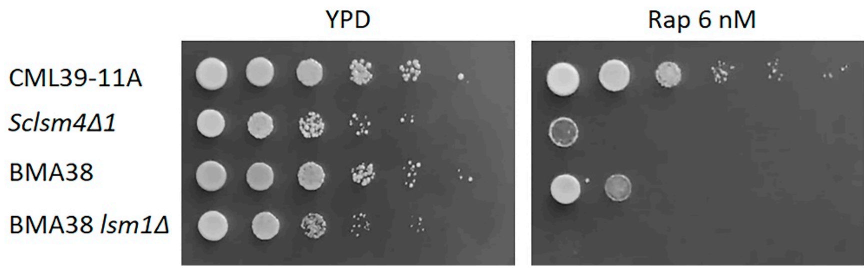

Figure S4. *Lsm1Δ* mutant shows high sensitivity to rapamycin at 6 nM, as demonstrated for the *Sclsm4Δ1* mutant. 10-fold dilution of MCY4 expressing the *Sclsm4Δ1* mutant, the *lsm1Δ* mutant, and their wild types (CML39-11A and BMA38) were spotted on YPD solid media containing 6 nM rapamycin and incubated at 28°C for 3 days. YPD was used as a growth control.

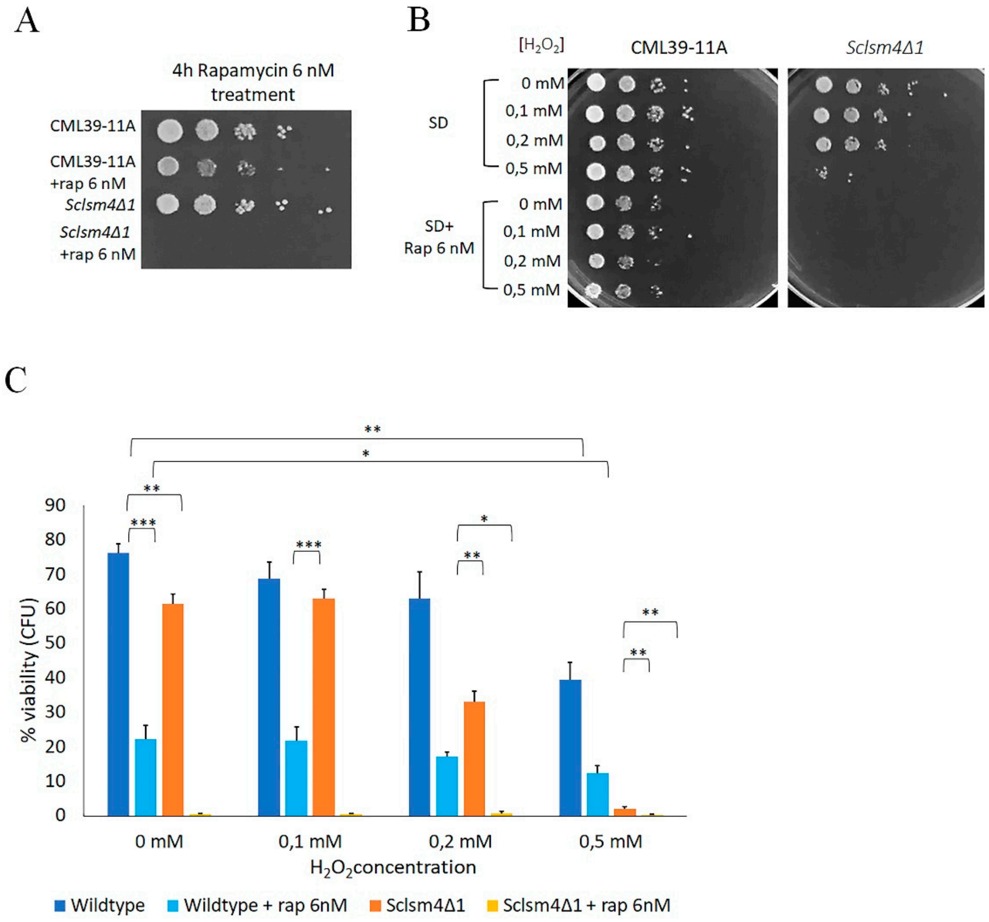

Figure S5. Treatment with a low dose of rapamycin does not protect the cells from oxidative stress**.** Cell viability of the CML39-11A (wild type) and *Sclsm4Δ1* mutant was measured after exposure to H_2O_2 at the indicated concentrations for 4 hours. 6 nM rapamycin was added 4 hour prior to exposure to H_2O_2. (A) 10-fold dilutions were spotted on complete solid media YPD after 4 hours of incubation in SD and SD + rapamycin 6 nM, and plates were incubated at 28°C for 3 days. (B) Treated and untreated

samples with rapamycin 6 nM were spotted in 10-fold dilution on complete solid media YPD after 4 hours of incubation with the indicated concentration of H_2O_2, and plates were incubated at 28°C for 3 days. (C) Cell viability was calculated as the percentage of microcolony forming cells. Data are represented as the mean of three independent experiments ± standard deviation. *p-value <0.05, **p-value <0.01***p-value<0.001.
